# Enhanced antioxidant, tyrosinase inhibition, and anti-inflammatory activities of *Praeparatum mungo* and three of its derivatives

**DOI:** 10.1038/s41598-023-48428-3

**Published:** 2023-12-04

**Authors:** Tzu-Chin Chang, Jie-Ling Cao, Yung-Sheng Lin, Shu-Ling Huang

**Affiliations:** 1 Ph.D. Program in Materials and Chemical Engineering, National United University, Miaoli, 36063 Taiwan; 2 Department of Chemical Engineering, National United University, Miaoli, 36063 Taiwan

**Keywords:** Biotechnology, Plant sciences

## Abstract

The main objective of this study is to explore the functions of *Praeparatum mungo* (PM) and three of its derivatives, *Praeparatum mungo*/turmeric (PM/T), *Praeparatum mungo*/bromelain (PM/B), and *Praeparatum mungo*/inorganic elements (PM/IE). The results indicated that additives included in the fermentation process of PM enhanced PM’s antioxidant properties. PM/B exhibited the highest total phenolic content (19.18 ± 0.46 mg gallic acid equivalent/g), DPPH free radical scavenging activity, and ferric reducing power. PM/IE exhibited the highest ABTS free radical scavenging activity and chelating ferrous ion activity. PM/T exhibited the best inhibitory tyrosinase activity. The 625 μg/mL PM extract can extensively reduce nitric oxide production of RAW264.7 macrophages stimulated by 1 μg/mL LPS and exhibited no cytotoxicity for anti-inflammatory applications. Additives in PM natural fermentation process can enhance antioxidant, tyrosinase inhibition, and anti-inflammatory properties of PM for future applications.

## Introduction

Advances in medicine have gradually allowed us to keep infectious diseases under control, but face age-related diseases remain a problem^1^. Unpaired electrons are unstable and capture electrons from other substances due to the presence of free radicals in the human body. This process causes a series of chain reactions that result in aging, disease, and other health problems^2,3^. Natural foods have been attracting attention because of their ability to replace antioxidants through chemical synthesis^4–6^.

Since antiquity, mung beans have been used as a form of traditional medicine. According to the "Compendium of Materia Medica" report, mung beans contain natural antioxidants, such as flavonoids^7^ and phenolic compounds^8^. They can lower blood lipid levels and have antioxidation, antibacterial, diuretic and detoxification functions^9^. Mung bean extract also has a hepatoprotective effect on acetaminophen-induced hepatotoxicity^10^.

*Praeparatum mungo* (PM), also called Lu-doh-huang in traditional Chinese medicine, is made through natural fermentation of mung beans. PM can enhance the functional compositions of mung beans^11,12^. PM has antioxidation and anti-inflammatory effects and can reduce the risk of liver tumors due to both the inhibition of lipid peroxidation and increase of antioxidant activity^13,14^. Besides, PM promotes the growth of skin tissues and suppresses the proliferation of A375 cells^15^. It can also inhibit tyrosinase activity and be applied in the development of tyrosinase inhibitors for inhibition of melanin biosynthesis^15^.

Additional ingredients can be added to the PM natural fermentation process^14^. Different additives in the fermentation process bring about different components and properties of PM. To the best of our knowledge, very little information about comprehensive comparison among PM and PM co-fermentation is available. To get a clear understand about PM functions, this study examined the antioxidant, tyrosinase inhibition, and anti-inflammatory properties of PM with and without three different additives for applications in resisting aging and disease.

## Materials and methods

### Sample preparation and extraction

PM was obtained using pure *Antrodia cinnamomea* through the natural solid-state fermentation of mung beans (*Vigna radiata* L. (Wilczek) cv. Tainan No. 5) purchased from Farmers' Association, Puzi City, Chiayi County, Taiwan. To obtain three derivatives of PM, turmeric, bromelain, and inorganic elements containing CaCO_3_ and MgO were added to the fermentation process, resulting in *Praeparatum mungo*/turmeric (PM/T), *Praeparatum mungo*/bromelain (PM/B), and *Praeparatum mungo*/inorganic elements (PM/IE), respectively. In the solid-state fermentation process, 83% mung bean and 17% additive were used. All the examined samples in this study, including PM, PM/T, PM/B, and PM/IE, were provided and preserved for future reference by Guan-Chang Biotechnology, Chiayi County, Taiwan. All local, national or international guidelines and legislation were adhered to in the production of this study.

To extract more water-soluble components to enhance antioxidant activities, the sample powder (10g) was soaked in deionized water (200 mL) then extracted under hot reflux (80 °C) for 2 h. As for non-water-soluble components, ethanol extraction was applied and optimized by the Taguchi design method according to total flavonoids which is approximately one-half to two-thirds of phenolic compounds in phytochemical substances^16,17^. The extract solution was then filtered and freeze-dried to preserve the quality of the specimen. Finally, all extracts were stored in a refrigerator (4 °C) before use in all experiments. To ensure the stability of antioxidant, tyrosinase inhibition, and anti-inflammatory activities of all extracts, the storage time was less than one week. In this study, water extract was used in the experiments of total phenolic content (TPC), antioxidant activity, and tyrosinase inhibition activity, while ethanol extract in total flavonoid content (TFC) and anti-inflammatory activity.

### Total flavonoid content

The Taguchi design method was used to determine the key extraction parameter^18^ for the highest TFC. In this study, the L_9_ (3^4^) of orthogonal table with three levels of four factors was selected (Table 1). The PM (10g) soaked in ethanol was extracted under hot reflux. After filtration, the organic solvent was removed with a rotary evaporation. Finally, all extracts were freeze-dried and stored at 4 °C until analyzed.

**Table 1:** Orthogonal table L9 (3^4^) in Taguchi design.

No	Time (min)	Temperature (℃)	Solvent concentration(%v/v)	Solid–liquid ratio
1	60	50	50	1: 10
2	60	60	70	1: 20
3	60	70	95	1: 30
4	90	50	70	1: 30
5	90	60	50	1: 10
6	90	70	95	1: 20
7	120	50	95	1: 20
8	120	60	50	1: 30
9	120	70	70	1: 10

The extract dissolved in dimethyl sulfoxide (50 μL, 50%) was mixed with CH_3_OH (50 μL) and NaNO_2_ (50 μL, 5%). After 5 min, AlCl_3_ (10 μL, 10%) was added, and the mixture was incubated at room temperature for 6 min. NaOH (100 μL, 1N) was then added and incubated at room temperature for 1 h. Finally, the absorbance of this mixture was measured at 510 nm. Quercetin was used as a standard, and the absorbance value of the sample was converted to the relative quercetin content and expressed as quercetin equivalent (QE).

### Total phenolic content

TPC was determined with the Folin-Ciocalteu's phenol reagent (FCP) method^19,20^. The extract was dissolved in deionized water (200 μL) and mixed with FCP (200 μL, 0.5N) in an Eppendorf tube to which Na_2_CO_3_ solution (200 μL, 10%) and deionized water (400 μL) were added. The mixture was incubated at room temperature for 1 h in the dark, and then centrifuged at 3000 rpm for 15 min. The supernatant (100 μL) was transferred to a 96-well plate and absorbance was measured at 730 nm. Gallic acid was used as a standard. The absorption value of the sample was substituted for the standard calibration curve to convert the relative total phenol content and express it as mg gallic acid equivalent (GAE) per gram of extract in dry weight (mg GAE/g).

## Antioxidant activity

### DPPH free radical scavenging activity

DPPH (1,1-diphenyl-2-picrylhydrazyl) free radical scavenging activity was determined using a previously developed method^21,22^. The extract dissolved in deionized water (100 μL) and the DPPH (100 μL, 160 mM) dissolved in 95% ethanol were mixed in a 96-well plate. After being incubated at room temperature for 30 min in the dark, the absorbance at 517 nm was measured. Vitamin C was used as a standard. The scavenging activity of DPPH free radical was calculated by the following equation.

$$DPPH \, scavenging \, activity \, \% \, = \,\left( {A_{Sample, \, 517 \, nm} {-}A_{Control, \, 517 \, nm} } \right)/A_{Sample, \, 517 \, nm} \, \times \,100\%$$

### ABTS free radical scavenging activity

ABTS (2,2'-azino-bis(3-ethylbenzothiazoline-6-sulfonic acid) cationic free radical scavenging activity was determined using a previously developed method^23,24^. The ABTS solution (1 mL, 7 mM) and K_2_S_2_O_8_ (1 mL, 2.45 mM) were mixed in an Eppendorf tube and then incubated at 4 °C for 16 h in the dark to generate a blue-green ABTS free radical solution; the resulting solution was diluted with 95% ethanol to achieve a background absorbance of 0.7 ± 0.05. The extract was dissolved in deionized water (80 μL), and the blue-green ABTS free radical solution were mixed in an Eppendorf tube. The mixture solution was incubated at room temperature for 10 min in the dark, and absorbance was measured at 734 nm. Trolox was used as a standard. The scavenging activity of ABTS free radical was calculated using the following equation

$$ABTS \, scavenging \, activity \, \% = \, \left( {A_{Sample, \, 734 \, nm} {-} \, A_{Control, \, 734 \, nm} } \right)/A_{Sample, \, 734 \, nm} {\times} 100 \, \%$$

### Chelating activity of ferrous ions

The chelating activity of ferrous ions was determined using a method developed in previous reports^25–27^. The extract dissolved in deionized water (800 μL) and FeSO_4_ (200 μL) were mixed in an Eppendorf tube. The mixture was incubated at room temperature for 5 min in the dark and then ferrozine (25 μL) was added. After 10 min, the mixture was centrifuged at 3000 rpm for 5 min, and absorbance was measured at 562 nm. EDTA-2Na was used as a standard, and the chelating activity of the sample relative to the EDTA-2Na was converted.

### Ferric reducing power

Ferric reducing power was determined using an assay developed in previous studies^28–30^. The extract dissolved in deionized water (200 μL), and phosphate buffer solution (200 μL, 0.2 M, pH 6.6) and potassium ferricyanide (200 μL, 10%) were mixed and incubated at 50 °C for 20 min. The trichloroacetic acid solution (200 μL, 10%) was added, and the resulting mixture was centrifuged at 3000 rpm for 10 min. The supernatant (400 μL) was then mixed with deionized water (400 μL) and ferric chloride solution (80 μL, 0.1%). After 10 min, its absorbance was measured at 734 nm. Butylhydroxyanisole (BHA) was used as a standard.

## Tyrosinase inhibition activity

The inhibition of tyrosinase activity was determined using a previously developed method^31–33^. The extract dissolved in deionized water (20 μL), tyrosinase solution (20 μL, 200 units/reaction), L-Dopa (40 μL, 3 mM) and phosphate buffer solution (100 μL, 67 mM) were mixed and incubated at 37 °C for 15 min in the dark, and the absorbance of this mixture was measured at 490 nm. Vitamin C was chosen as a standard, and the inhibition of tyrosinase activity was calculated using the following equation:

$$Tyrosinase \; inhibition \; activity\% = \, \left( {A_{Sample, \, 490 \, nm} {-} \, A_{Control, \, 490 \, nm} } \right)/A_{Sample, \, 490 \, nm} {\times} 100 \, \%$$

## Anti-inflammatory activity

### Cell cultivation

Cell cultivation in this study was conducted using a previously developed method^34,35^. The mouse macrophage (RAW264.7) cells were incubated in DMEM containing 10% fetal bovine serum and 1X P/S antibiotic solution; the culture conditions were 37 °C and 5% CO_2_. The cells were subcultured in T-25 flasks under a microscope.

### Cytotoxicity

This study used a previously developed MTT method to measure cytotoxicity^36,37^. RAW264.7 cells (1 × 10^4^ cells/well) were attached to a 96-well plate containing batches of 100 μL medium with different sample concentrations and incubated at 37 °C and 5% CO_2_ for 24 h. The medium was then removed, and MTT reagent (100 μL, 0.5 mg / mL) was added. After 3 h, the MTT reagent was removed, and DMSO (100 μL, 100%) was added to dissolve the purple crystals. Finally, it was incubated for 30 min and, its absorbance measured at 570 nm.

### Nitric oxide content

Nitric oxide (NO) content was determined using a previously developed method^38,39^. RAW264.7 cells (1 × 10^5^ cells/well) was attached to a 96-well plate, and batches of the medium (200 μL) containing 1 μg/mL LPS and different sample concentrations were added and incubated at 37 °C and 5% CO_2_ for 24 h. The cell culture supernatant was then mixed with Griess reagent, and its absorbance was measured at 530 nm. Sodium nitrite was used as a standard.

## Results and discussion

### Total flavonoids

Flavonoids are a group of polyphenolic compounds and the main biological activity of flavonoids is their antioxidant activity^40^. For phytochemical substances, one-half to two-thirds of phenolic compounds are flavonoids^16,17^. Therefore, flavonoids are significantly correlated with antioxidant activity^41^. Flavonoids can function as potent antioxidants to reduce and prevent inflammation^42–44^. Table 2 shows that PM extract had flavonoids between 26 and 36 μg QE/g, depending on extraction conditions. The extraction solution in the No. 1 condition—60-min extraction, 50 °C, 50% ethanol, and solid–liquid ratio of 1:10—had the highest TFC.

**Table 2:** TFC of PM extract under different extraction conditions.

No	Time (min)	Temp. (℃)	Solvent concentration (v/v %)	Solid–liquid ratio	TFC (μg QE/g)
1	60	50	50	1: 10	35.86 ± 0.15
2	60	60	70	1: 20	33.85 ± 0.34
3	60	70	95	1: 30	26.76 ± 0.03
4	90	50	70	1: 30	30.99 ± 0.24
5	90	60	50	1: 10	26.04 ± 0.16
6	90	70	95	1: 20	34.86 ± 0.36
7	120	50	95	1: 20	26.41 ± 0.05
8	120	60	50	1: 30	35.42 ± 0.05
9	120	70	70	1: 10	33.73 ± 0.33

The Taguchi experiment design was used to evaluate the extraction efficiency of total flavonoids from PM under various conditions. A higher delta value indicates better performance, representing the larger signal-to-noise ratio effect of the four factors in this study^45,46^. Table 3 shows the delta value in the order of solvent concentration > extraction time > solid–liquid ratio > extraction temperature. The main influencing factor of PM extraction in this study was the solvent concentration.

**Table 3:** Signal-to-noise ratio response table of TFC for extracting PM by various factors.

Level	Time (min)	Temp. (℃)	Solvent concentration (v/v %)	Solid–liquid ratio
1	30.08	29.78	30.97	29.99
2	29.66	29.96	30.32	29.96
3	29.99	29.98	28.43	29.79
Delta	0.42	0.20	2.54	0.20
Rank	2	4	1	3

## Total phenolic content

**Figure 1:**
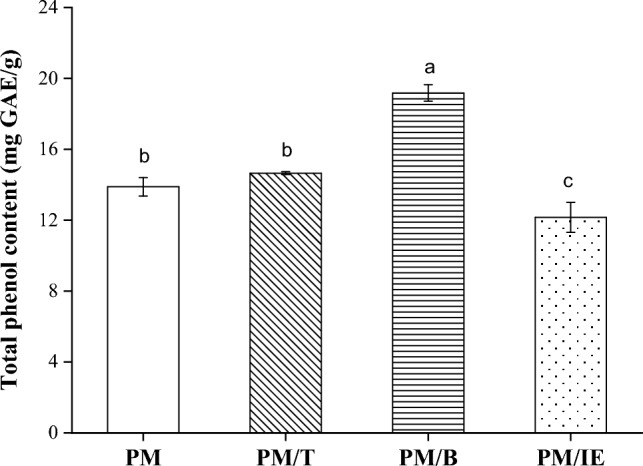
Total phenol content of water extracts in this study. Mean with the same lowercase letters are not significantly different at the 5% level, according to Fisher’s protected least significant difference test.

Polyphenols scavenge free radicals through their antioxidant activity. The total phenol content is often regarded as an antioxidant capacity because a higher phenol content indicates more antioxidant components. The antioxidant capacity of samples extracted with deionized water is shown in Fig. 1. PM/B has the highest total phenolic content (19.18 ± 0.46 mg GAE/g). In this study, the total phenolic content of PM (13.88 ± 0.53 mg GAE/g) was higher than the reported value of mung beans (9.94 ± 0.18 mg GAE/g)^3^. The solid-state fermentation of mung beans can effectively increase the total phenol content and number of antioxidant components. Among the fermentation varieties, the samples of PM/IE had the lowest total phenol content. Inorganic element components of PM/IE do not extensively increase the content of polyphenol substances.

## Antioxidant activity

**Figure 2:**
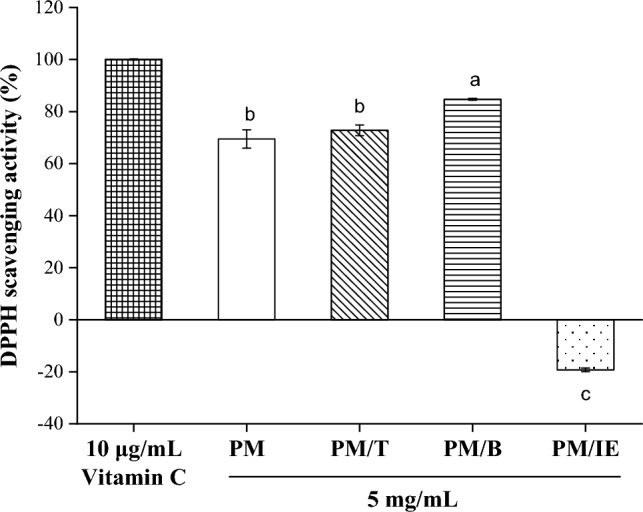
DPPH free radical scavenging activity of 10 μg/mL vitamin C and 5 mg/mL water extracts (PM, PM/T, PM/B, PM/IE). Mean with the same lowercase letters are not significantly different at the 5% level, according to Fisher’s protected least significant difference test.

The free radical reaction initiates the accelerated oxidative degradation of lipids, which has negative health outcomes^47,48^. Antioxidants can block free radicals, such as DPPH free radical, ABTS free radical, superoxide anion radicals or hydroxyl radicals. The DPPH free radical scavenging determination method is stable and commonly used by researchers^49^. In this study, vitamin C (10 μg/mL) was used as a standard for DPPH free radical scavenging activity; the scavenging activities of water extract samples (5 mg/mL) were compared with the activity of vitamin C. PM, PM/T and PM/B had DPPH free radical scavenging activity (Fig. 2), and PM/B had the best performance among them. This result agrees with the previous report that PM with traditional Chinese medicine formulas had DPPH free radical scavenging activity^50^. The metal ions of PM/IE in the determination system slow down the kinetic processes underlying DPPH free radical scavenging because DPPH free radicals are able to form complexes with metal ions^51^. Therefore, PM/IE does not conform to the mechanism of DPPH free radical scavenging.

**Figure 3:**
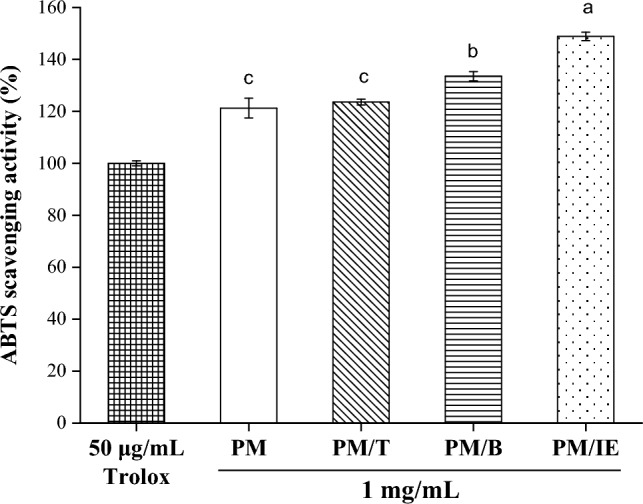
ABTS free radical scavenging activity of 50 μg/mL Trolox and 1 mg/mL water extracts (PM, PM/T, PM/B, PM/IE). Mean with the same lowercase letters are not significantly different at the 5% level, according to Fisher’s protected least significant difference test.

The determination of antioxidant capacity also includes ABTS cationic free radical scavenging. The antioxidants can provide electron reducible ABTS cationic free radicals to assess scavenging activity. The ABTS free radical scavenging activity of Trolox (50 μg/mL) was used as a standard and compared with the scavenging activity of water extract samples (1 mg/mL). Figure 3 shows that all four water extract samples exhibited ABTS cationic radical scavenging activity, and PM/IE exhibited the highest scavenging activity. Thus, adding ingredients to PM can enhance ABTS free radical scavenging activity.

**Figure 4:**
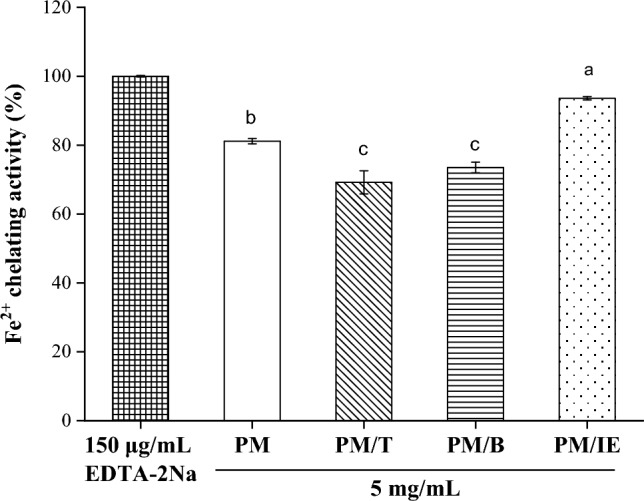
Chelating ferrous ion activity of 150 μg/mL EDTA-2Na and 5 mg/mL water extracts (PM, PM/T, PM/B, PM/IE). Mean with the same lowercase letters are not significantly different at the 5% level, according to Fisher’s protected least significant difference test.

Metals or their ions are ubiquitous and can have catalytic oxidation effects. Among them, Fe^2+^ is an influential oxidant that causes a free radical chain reaction; it can promote the automatic oxidation of lipids and induce or catalyze reaction^52^. EDTA-2Na (150 μg/mL) was applied to be 100% chelating Fe^2+^ activity, and the water extracts (5 mg/mL) of the samples were compared. Figure 4 shows that the four PM samples chelated Fe^2+^, and PM/IE did so most vigorously.

**Figure 5:**
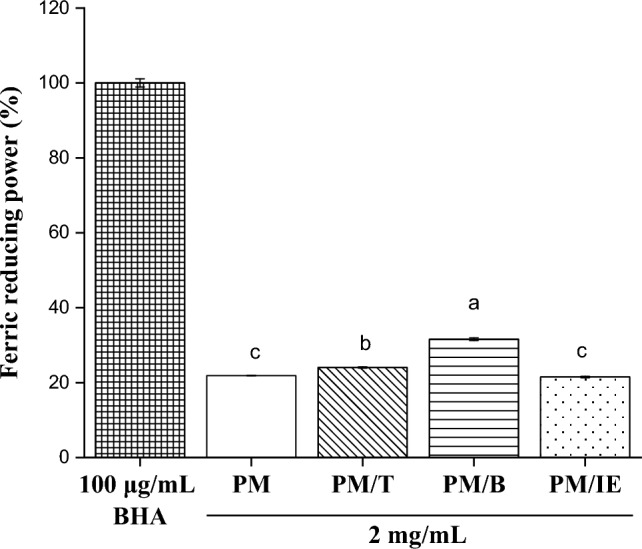
Ferric reducing power of 100 μg/mL BHA and 2 mg/mL water extracts (PM, PM/T, PM/B, PM/IE). Mean with the same lowercase letters are not significantly different at the 5% level, according to Fisher’s protected least significant difference test.

**Figure 6:**
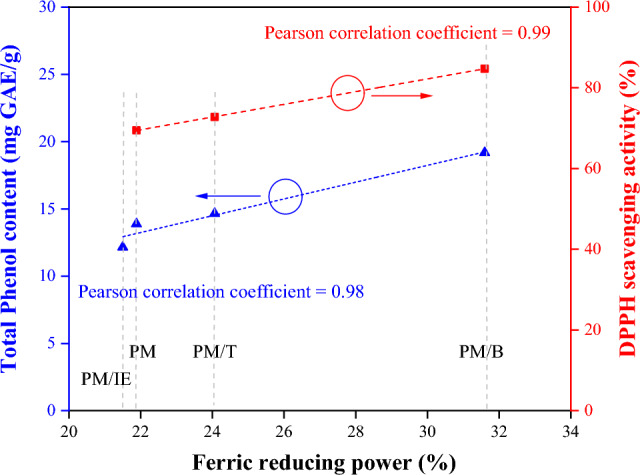
Correlation analysis between ferric reducing power and total phenol content/DPPH free radical scavenging activity.

Antioxidant have ferric reducing power and can provide electrons to reduced Fe^3+^ to Fe^2+^^53^; ferric reducing power can thus be used as an indicator of antioxidant capacity. The ferric reducing power from applying BHA (100 μg/mL) at 100% was compared with the ferric reducing power in water extract samples (2 mg/mL). Figure 5 shows that four PM samples exhibited ferric reducing ability. The PM/B sample exhibited a higher ferric reducing power value than other samples. The results are positively correlated with the previous total phenol content and DPPH free radical scavenging activity (Fig. 6).

## Tyrosinase inhibition activity

**Figure 7:**
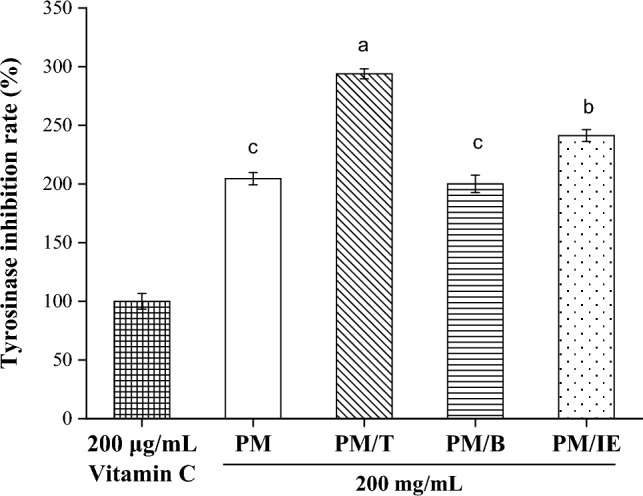
Tyrosinase inhibition activity of 200 μg/mL vitamin C and 200 mg/mL extracts (PM, PM/T, PM/B, PM/IE). Mean with the same lowercase letters are not significantly different at the 5% level, according to Fisher’s protected least significant difference test.

Tyrosinase is a copper-containing enzyme^54^ that is key in catalyzing melanin production^55^, which can cause health problems at abnormal levels^56^. The activity to inhibit tyrosinase was determined using PM extract in this study. A sample in which vitamin C (200 μg/mL) was the tyrosinase inhibition standard was compared with four PM extract samples (200 mg/mL) (Fig. 7). PM with turmeric or inorganic elements has a higher activity to inhibit tyrosinase than pure PM, and the PM/T has the highest inhibitory activity. These results agree with the previous report that PM with traditional Chinese medicine formulas can inhibit tyrosinase activity and tyrosinase inhibition increases with PM concentrations^50^.

## Anti-inflammatory activity

### Cell viability

**Figure 8:**
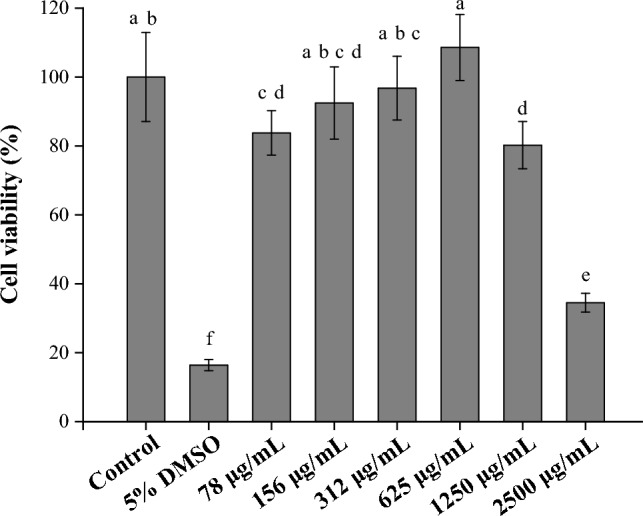
Effect of PM extract concentration on the cell viability of RAW264.7. Mean with the same lowercase letters are not significantly different at the 5% level, according to Fisher’s protected least significant difference test.

MTT is a common method for measuring cytotoxicity^57^. The succinate dehydrogenase in living cells can be converted to purple crystal by MTT reagent. A higher succinate dehydrogenase activity occured under the condition of more cells surviving when RAW264.7 is cultured in the tested sample. As shown in Fig. 8, the control group was cultured with cells and medium but not cultured with PM extract; the experimental groups had different concentrations (78, 156, 312, 625, 1250, and 2500 μg/mL) of PM extract applied to them. When the cell viability of the control group was set to 100%, the concentration of PM extract below 1250 μg/mL was considered a viable admissible concentration. Besides, PM below 625 μg/mL could increase the cell proliferation rate in a dose-dependent manner which corresponding to the finding of a previous literature^15^.

### Nitric oxide content

**Figure 9:**
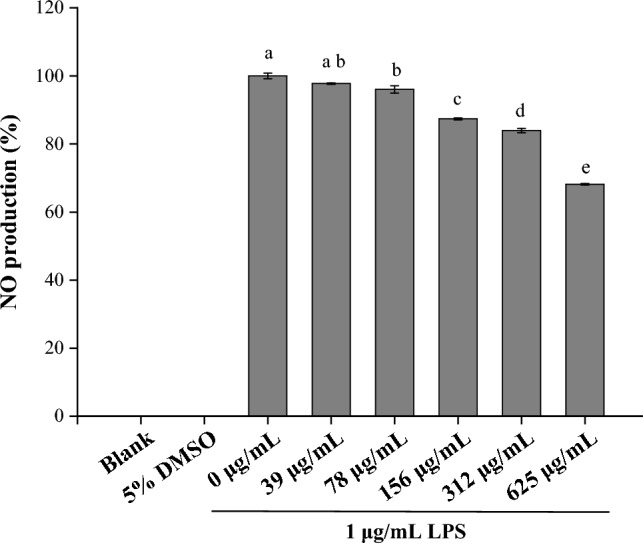
Nitric oxide production by RAW264.7 under different concentrations of PM extract containing 1 μg/mL LPS. Mean with the same lowercase letters are not significantly different at the 5% level, according to Fisher’s protected least significant difference test.

The inflammatory response is a defensive response that causes inflammation through physiological behavior^58^. Long-term inflammation will damage human organs^59^ and cause many diseases^60^. In this study, PM extract was used to test the activation of RAW 264.7 by LPS^61^. As shown in Fig. 9, both blank and 5% DMSO groups cultured without LPS and PM extract did not exhibit NO production. The other groups were cultured with various concentrations (0, 39, 78, 156, 312 and 625 μg/mL) of PM extract and 1 μg/mL of LPS. PM extract concentration is negatively correlated with NO production. Per restrictions on cytotoxicity, PM can be used at a concentration of 625 μg/mL to inhibit NO production for anti-inflammation applications.

## Conclusion

This study successfully demonstrated that PM fermented with additives can enhance several antioxidant functions. PM with bromelain can enrich total phenolic content, DPPH free radical scavenging activity, and ferric reducing power. PM with inorganic elements improves ABTS free radical scavenging activity and chelating ferrous ions activity. PM with turmeric has the best inhibitory tyrosinase effect. PM extract with concentration of 625 μg/mL can greatly reduce LPS-stimulated macrophage activation for NO production, and exhibits no cytotoxicity for anti-inflammatory applications. PM fermented with additives can be used for future applications such as functional food or cosmetic formulations.

## Data Availability

Correspondence and requests for materials should be addressed to Y.S.L. and S.L.H.
